# Characterization of Electrospun Poly(ε-caprolactone) Nano/Micro Fibrous Membrane as Scaffolds in Tissue Engineering: Effects of the Type of Collector Used

**DOI:** 10.3390/membranes12060563

**Published:** 2022-05-28

**Authors:** Dianney Clavijo-Grimaldo, Ciro Alfonso Casadiego-Torrado, Juan Villalobos-Elías, Adolfo Ocampo-Páramo, Magreth Torres-Parada

**Affiliations:** 1 School of Medicine, Universidad Nacional de Colombia, Bogotá 111321, Colombia; jvillalobos@unal.edu.co (J.V.-E.); admocampopa@unal.edu.co (A.O.-P.); mtorrespa@unal.edu.co (M.T.-P.); 2 School of Medicine, Fundación Universitaria Sanitas, Bogotá 111321, Colombia; ccasadie@gmail.com

**Keywords:** electrospinning, polycaprolactone, scaffold, tissue engineering

## Abstract

Electrospinning is an electrohydrodynamic technique that transforms a polymer solution into nano/microscopic diameter fibers under the influence of a high-voltage electric field. Its use in the fabrication of nano/micro fibrous membranes as scaffolds for tissue engineering has increased rapidly in recent years due to its efficiency and reproducibility. The objective of this study is to show how the use of the same polymeric solution (polycaprolactone 9% *w*/*v* in chloroform: isopropanol 50:50) and identical electrohydrodynamic deposition parameters produces fibers with different characteristics using a flat collector platform with movements in the X and Y axes vs. a conventional rotary collector. The manufactured nano/microfibers show significant differences in most of their characteristics (morphology, roughness, hydrophilicity, and mechanical properties). Regarding the diameter and porosity of the fibers, the results were similar. Given that scaffolds must be designed to guarantee adequate survival and the proliferation and migration of a certain cell type, in this study we analyze how the variations in the characteristics of the fibers obtained are essential to defining their potential application.

## 1. Introduction

The low availability of donors and the morbidity associated with transplants mean that ET is considered a useful strategy to restore or reestablish the function of pathologically altered tissues and organs. The tissue engineering (TE) approach involves the regeneration of tissue on a suitable support and implanting it at the goal site. Tissue regeneration functionally requires a microenvironment that mimics the original site to obtain an adequate cellular response and to provide optimal conditions for regeneration. Traditionally, TE is defined as “the body persuasion of to re-pair itself, releasing, at the appropriate sites, molecular signals, cells and/or supporting structures”. It seeks to manufacture tissues, directing molecular and mechanical signals to specific cells to restore or reestablish normal function [1]. TE uses three basic components, cells, scaffolds, and a chemical environment (hormones, growth factors, etc.). The scaffolds’ role is to mimic the extracellular matrix (ECM), a multiphase nano/microstructure material that is essential for cell viability, and to maintain the morphological, mechanical, and functional characteristics of the tissue. Several strategies are used in TE. In one of them, the construct (cells + scaffold + growth factors) is placed in a bioreactor that reconstructs the designed tissue in vitro. Another strategy is the implantation of the scaffold in the patient so that it fulfills its regenerative role in vivo, The first strategy is known as TE in vitro (or ex vivo) and the second TE in vivo (or in situ). Bioreactors are used to mimic in vitro environmental conditions in vivo and/or to provide the chemical environment that regulates cell proliferation and differentiation and the production of ECM prior to implantation in vivo [2,3]. Nano/microfibrous polymeric membranes have been widely used as biomaterials for the fabrication of scaffolds in TE due to their properties (biocompatibility, high surface-to-volume ratio, high porosity, biodegradation, and mechanical properties). The most used natural polymers for the manufacture of scaffolds are silk, collagen, gelatin, fibrinogen, alginate, and chitosan. Among the most used synthetic polymers are polycaprolactone (PCL), poly(lactic acid), polyglycolide and poly(l-lactide-co-glycolide), among others [4].

Ideally, nano/microfibrous polymeric membrane used as scaffold should have the following characteristics: biocompatibility and nontoxicity properties, a three-dimensional structure, high porosity, it should be biodegradable or bioresorbable, have controllable degradation and reabsorption rates, a chemically appropriate surface to promote proliferation and cell differentiation, hierarchical organization, and mechanical properties similar to the tissue where it will be implanted. Additionally, the technique with which it is manufactured must be versatile and easily controllable and reproducible [5,6]. However, some of the scaffolds used have shown some limitations, such as inadequate physicochemical and mechanical properties, as well as inappropriate porosity, wettability, alignment, roughness, and surface-to-volume ratios, which leads to insufficient tissue regeneration. Therefore, there is a need to explore techniques and obtain materials for scaffold manufacturing that mimic and simulate the structural, topographic, mechanical, and conductive properties of a specific ECM to promote the regeneration of the tissue function [7,8,9,10,11,12,13].

Numerous techniques have been used to manufacture scaffolding: freeze-drying, thermal-induced phase separation, gas foaming, rapid prototyping, stereolithography, fused deposition modeling, selective laser sintering, three-dimensional printing, bioprinting, etc. [14]. However, these systems have drawbacks, such as their limited print resolution, in which cells cannot be formed and organized precisely. Another drawback is the solidification and gelation requirements during the printing process, which limit the materials (hydrogels) that can be used [15]. Indeed, it is necessary to rely on other systems and methods to complement and improve the efficiency, survival, and proliferation of cells. Electrohydrodynamic techniques improve the properties of materials and devices. Due to the simplicity and flexibility of the experimental setup of these techniques, they have been used successfully in the fabrication of particulate materials with controllable compositions, structures, sizes, morphologies, and shapes. These attributes, in addition to not having as many limitations in the working materials, make electrohydrodynamic techniques an extraordinary tool for preparing and assembling a wide range of micro- and nanostructured materials [16,17]. Electrospinning, in which a polymer in solution is subjected to a high voltage to produce nano/microfibers, is one of the most widely used techniques today due to its cost, ease of manufacture, low material requirements, and the evaporation of the solvents used, reducing the risks of toxicity inherent in some solvents and favoring greater biocompatibility. By adjusting variables such us the diameter of the fibers (and therefore the surface-to-volume ratio), the surface roughness of the fibers, and the porosity, the interconnectivity of the pores and mechanical properties of the scaffolds can be controlled to adjust them to specific conditions (bone regeneration, heart, skin, etc.). The main variables considered depend on the characteristics of the polymer solution (the molecular weight of the polymer, solvent, surface tension, concentration, and the viscosity of the solution, among others); the process (solution flow, the voltage applied between the needle through which the solution passes and the collector, the type of collector, needle–collector distance); and the environment (temperature and humidity) [11,13,14,18]. In addition, the fibers can be enriched with drugs and/or growth factors so that the scaffold works as a release system that favors or controls specific cellular functions, contributes to the management of pathologies, modulates the response of the immune system towards the biomaterial and, in general, increases its bioactivity [19,20,21].

The effect that the collector has on the evaporation of the solvent and on the orientation, diameter, density, fiber–fiber junction points, and mechanical properties of the fibers obtained is known. The rotary collector (RC) is the approach most frequently used in TE applications. There are numerous studies on how the diameter and rotation speed can determine the type of fibers obtained, and the changes that occur in them when compared to those obtained with flat collectors [22,23,24,25]. More recently, collectors have been adapted to combine electrospinning principles with 3D printing. In this sense, the 3D configuration depends on the deposition time and the displacement of the collector used, since it can be designed to move in several axes, with the aim of modifying certain characteristics (for example, tensile strength and elasticity) [26,27,28,29]. The effect on fiber characteristics of using an RC versus the configuration of the flat collector on a moving platform in the X–Y axes has not been compared and analyzed in detail.

In this study, PCL fiber scaffolds were constructed in a known standard solution [30]. The fibers were collected in two types of collectors: a conventional RC and a flat collector platform that moved in two axes (XYP) to compare the diameter and roughness of the individual fibers and the mechanical properties, porosity, and hydrophilicity of the fibers.

## 2. Materials and Methods

### 2.1. Materials

PCL (CAS # 134490-19-0 and average Mn = 80.000 g/mol), chloroform (99.5%, CAS # 67-66-3 and Mn = 119.38 g/mol), and isopropyl alcohol (99.7% CAS # 67-66-3 and Mn = 60 g/mol) were used. All the chemical agents used were supplied by Sigma-Aldrich (San Luis, MO, USA).

### 2.2. Preparation of the Solution

PCL was used in a 9% *w*/*v* solution in a 50:50 *v*/*v* mixture of chloroform and isopropyl alcohol. The resulting solution was stored at room temperature for 48 h and, prior to its use, it was subjected to homogenization using ultrasound (Model ATM40-2LCD, ATU UL-TRA-SON-IC) with a frequency of 50 Hz for 60 min at 17 °C.

### 2.3. Electrospinning Process

**Figure 1:**
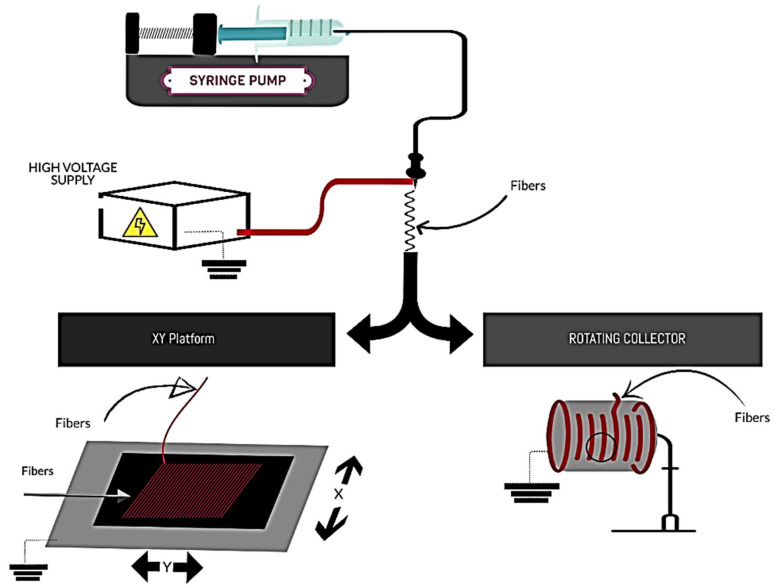
Schematic illustration of the electrospinning process performed: XY platform (flat collector platform with movements in the X and Y axes) vs rotary collector.

The scaffolds were developed using electrospinning equipment composed of a high-voltage source Model CZE1000R (Spellman high voltage corporation, Hauppauge, NY, USA), a dosing pump KDS 100 (KD Scientific Inc., Holliston, MA, USA), syringe and needle (Upchurch Scientific Inc., Oak Harbor, WA, USA), and two types of collectors: XYP (own manufacture) and RC ESD30s (Nanolab Instruments Sdn Bhd, Malaysia) (Figure 1). The definitive parameters were voltage (14 kV), distance between the needle tip and the collector (14 cm), solution flow (1 mL /h), and two deposition times (45 and 90 min). The collection of fibers was completed on aluminum sheets placed on the collector. A linear velocity was set in the XYP, in both axes, of 0.0025 m/s. To obtain equivalent parameters, the angular velocity of the RC (r: 0.04 m) was calculated based on the linear velocity of the platform, resulting in a speed of 6 rpm. The scaffolds produced were divided into four groups: scaffolds developed in the RC during 45 and 90 min of deposition (RC45 and RC90), and scaffolds developed in the XYP with equal deposition times (XYP45 and XYP90). The process was performed at 20 ± 2 °C room temperature and 60 ± 5% RH.

### 2.4. Roughness of the Individual Fibers

The surface roughness of the fibers was measured by means of atomic force microscopy AFM; (Asylum Research—MFP-3D-BIO (Oxford Instruments, Santa Barbara, CA, USA) in a sampling area of 1 × 1 µm^2^. The roughness values (root mean square (Rms) and arithmetic mean (Ra)) were calculated in triplicate using the open-source software Gwyddion, version 2.56. Since the objective was to determine the roughness of individual fibers (and not of the scaffold produced) and taking in count compression forces with longer deposition times, the fibers used in this test were collected 30 s after their deposition.

### 2.5. Mechanical Tests

Mechanical tests were performed on the four groups of scaffolds. The scaffolds were cut into rectangular samples of 10 mm × 110 mm with a thickness 0.0385 mm. The tensile strength testing of the samples was performed using a universal testing machine AG-IS 5KN, (Shimadzu corporation, Kyoto, Japan). The tests were carried out according to ASTM D882 with a preload of 0.003 N and at a speed of 20 mm/min at room temperature (20.8 °C) and 60% RH. The orientation of the samples for the mechanical tests was longitudinal. The tests were carried out in triplicate.

### 2.6. Morphology and Fibers Diameter

The fiber morphology of the four groups of the scaffolds was studied using high-vacuum scanning electron microscopy (SEM) Tescan Vega 3, (Tescan Analytics, Brno, Czech Republic) with an operating voltage of 10 kV and a magnification of 500× to find the areas of interest. The fiber diameter was analyzed using the public domain image analysis software Image J (National Institutes of Health) at a magnification of 5000×. Measurements were made before and after the mechanical tests.

### 2.7. Contact Angle of the Scaffolds

Contact angle tests, (ASTM D5725-99/2008, American Society for Testing and Materials, West Conshohocken, Pennsylvania, U.S) were performed on all four scaffold groups. Samples of 10 mm × 10 mm were placed on slides and 10 µL drops of deionized water were deposited on the surface. Images were obtained with a digital camera Canon eos rp, Lens: Canon rf 24–105 mm, f4 (Canon Inc., Melville, NY, USA) Contact angles were calculated using the public domain image analysis software Image J (National Institutes of Health).

### 2.8. Porosity Measurement of the Scaffolds

A methodology previously described in another study [31] was used due to its effectiveness in measuring various layers of nano/microfibers. Using the public domain image analysis software Image J, SEM images of the four groups of scaffolds were converted to binary images using three thresholds and the porosity of each scaffold was measured in three layers. Three thresholds were calculated to convert the original image to binary form based on the mean and standard deviation of the pixel values of the image across the equations: T1 = (µ + σ)/255, T2 = µ/255 and T3 = (µ − σ)/255, where µ and σ are the mean and the standard deviation of the image matrix, respectively. The percentage of porosity (P) of each binary image was obtained using the average intensity of the images, as indicated in the equation: P = (1 − n/N) * 100, where n is the number of white pixels and N is the total number of pixels in the total volume of the binary image [31]. Measurements were made before and after the mechanical tests.

### 2.9. Statistical Analysis

Variance analysis (ANOVA) and the F-test were performed to assess the amount of variability between group means in the context of within-group variation to determine if the mean differences were statistically significant. In this study, if the *p*-value was ≤0.05 and the calculated F-value was greater than the critical value F (the ratio of two variances), the differences were considered significant.

## 3. Results

### 3.1. Roughness of the Individual Fibers

**Figure 2:**
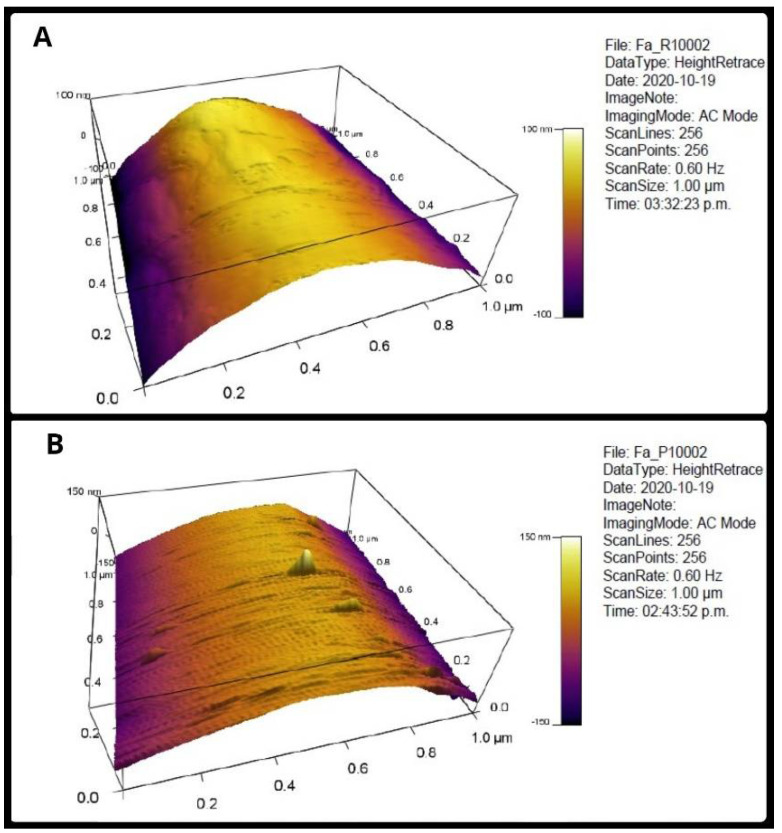
AFM of PCL fibers. (**A**) is for the RC scaffold, (**B**) for the XYP Scaffold. AFM: atomic force microscope, XYP: flat collector platform with movements in the X and Y axes, RC: rotary collector.

Figure 2 shows the appearance of a sector of fibers made on RC and XYP. Table 1 shows the Rm and Ra values obtained in the fibers, as well as their averages and standard deviation. The average Ra of the surface of the RC fibers was 42.72 ± 12.12 nm and the Rms was 51.47 ± 15.32 nm, whereas the surface roughness values for the XYP fibers decreased to 38.25 ± 23.7 nm and 36.94 ± 15.6 nm for Ra and Rms, respectively. Despite the differences in the averages of the RC fibers versus the XYP fibers, the analysis of variance did not show significant differences between the two groups either for Rm (*p* = 0.31, F = 1.32 and critical F = 7.71) or for Ra (*p* = 0.79, F = 0.08 and critical F = 7.71).

### 3.2. Mechanical Tests 

**Figure 3:**
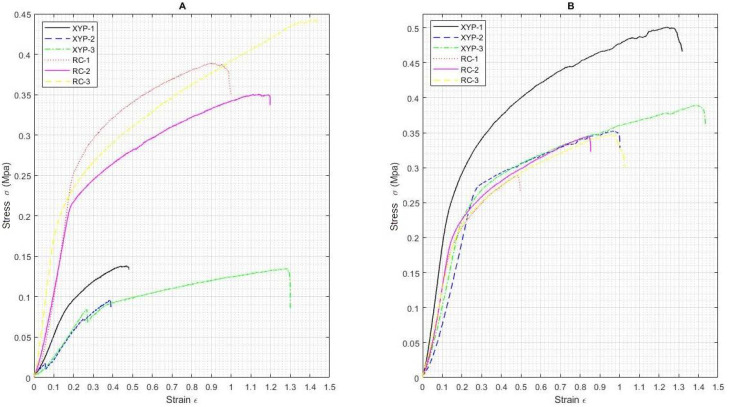
Stress-strain curves of PCL scaffolds obtained using RC and XYP. (**A**) Deposition time: 45 min, (**B**) deposition time: 90 min.

Figure 3 shows the stress-strain curves for the scaffolds obtained on RC and XYP with deposition times of 45 min (Figure 3A) and 90 min (Figure 3B).

Table 2 shows the stress and strain values for the scaffolds obtained on RC and XYP at 45 min and 90 min of deposition, as well as their arithmetic averages and standard deviation. For a deposition time of 45 min, the scaffold deposited on RC showed a tensile stress value of 0.39 ± 0.05 MPa, Young’s modulus of 1.11 MPa, and an elongation at break of 114.91% compared to the scaffold deposited in XYP, which showed lower average values, with a tensile stress value of 0.12 ± 0.02 MPa, Young’s modulus of 2.49 MPa, and an elongation at break of 70.98%. For a deposition time of 90 min, the scaffold deposited on RC showed a tensile stress value of 0.41 ± 0.08 MPa, Young’s modulus of 1.69 MPa, and an elongation at break of 119.88% compared to the scaffold deposited in XYP, which also showed lower average values: tensile stress of 0.33 ± 0.03 MPa, Young’s modulus of 1.24 MPa, and an elongation at break of 75.87%. A highly significant difference was observed between the maximum stress value for the RC scaffolds versus XYP scaffolds when the deposition time was 45 min (*p* = 0.0008, F = 81.55, and critical F = 7.71); however, no significant difference was observed for the elongation at break (*p* = 0.24, F = 1.89, and critical F = 7.71). For the deposition time of 90 min, no significant differences were observed either in the maximum stress value (*p* = 0.15, F = 3.24, and critical F = 7.71) or in the elongation at break (*p* = 0.07, F = 5.45, and critical F = 7.71).

### 3.3. Morphology and Fiber Diameter

**Figure 4:**
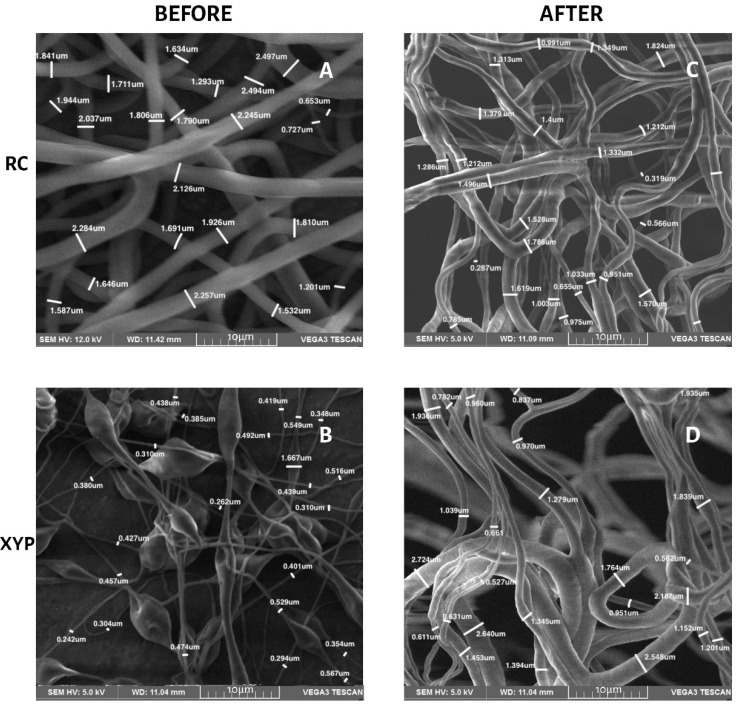
Fiber diameter in SEM micrographs, 45 min of deposition. (**A**) RC45 before mechanical tests, (**B**) XYP45 before mechanical tests, (**C**) RC45 after mechanical tests, (**D**) XYP45 after mechanical tests.

**Figure 5:**
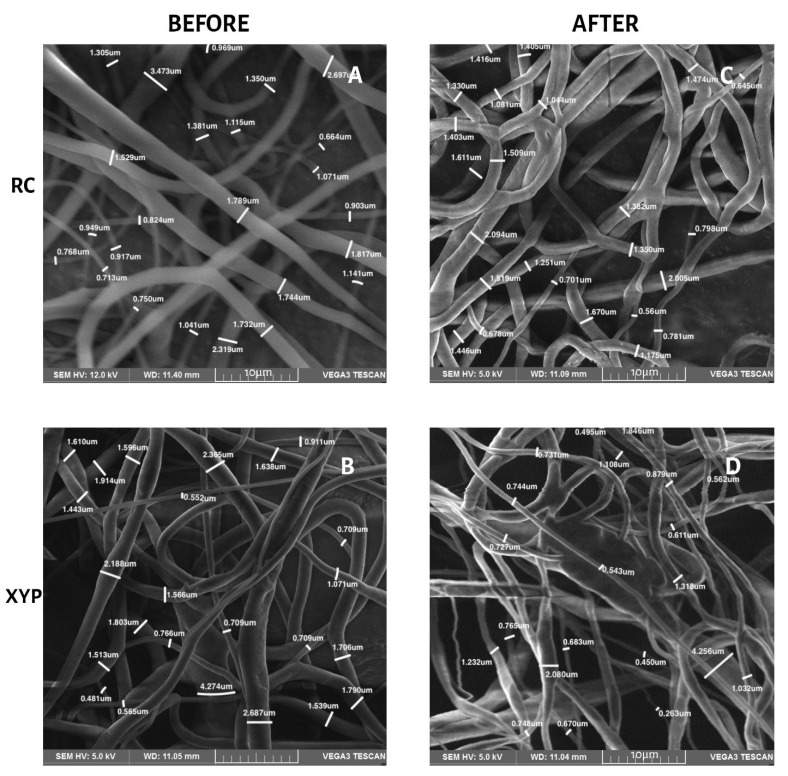
Fiber diameter in SEM micrographs, 90 min of deposition. (**A**) RC90 before mechanical tests, (**B**) XYP90 before mechanical tests, (**C**) RC90 after mechanical tests, (**D**) XYP90 after mechanical tests.

Table 3 shows the fiber diameters, their means and standard deviations for the four groups of scaffolds before and after the mechanical tests. No significant differences were found in the diameter of the fibers, according to the type of collector used, before the mechanical tests, or after 45 min of deposition (*p* = 0.24, F = 1.51, and critical F = 4.96) or 90 min of deposition (*p* = 0.47, F = 0.57, and critical F = 4.96). The differences are also not significant when comparing the type of collector at 45 min (*p* = 0.41, F = 0.74 and critical F = 4.96) and 90 min of deposition (*p* = 0.55, F = 0, 37 and critical F = 4.96) after mechanical tests. Figure 4 and Figure 5 show the SEM morphology for deposition times of 45 min (Figure 4) and 90 min (Figure 5) in each collector used. The orientation of the fibers in both types of collectors was observed randomly, without any predominant pattern. The fibers obtained with RC were more uniform and did not present pearls or beads, whereas those obtained with XYP presented numerous pearls and beads, especially with 45 min of deposition.

### 3.4. Contact Angle of the Scaffolds

The contact angle values obtained are shown in Table 4. The RC45 scaffolds had an average contact angle of 51.95° ± 1.8°. The RC90 scaffolds achieved a 1.2% increase in their contact angles (58.19° ± 6.6°). For XYP45 scaffolds there was a significant 55% increase compared to RC45 contact angles (80.52° ± 3.8°) and a 7% decrease compared to XPY90 scaffold contact angles (74.77° ± 15.6°). An increase in hydrophilicity was observed in RC45 scaffolds, with significant differences in the contact angle (*p* = 1.08^−5^, F = 178.77, and critical F = 5.98) when compared to XYP45 scaffolds. No significant differences were observed between RC90 versus XYP90 scaffolds.

### 3.5. Porosity Measurement

**Figure 6:**
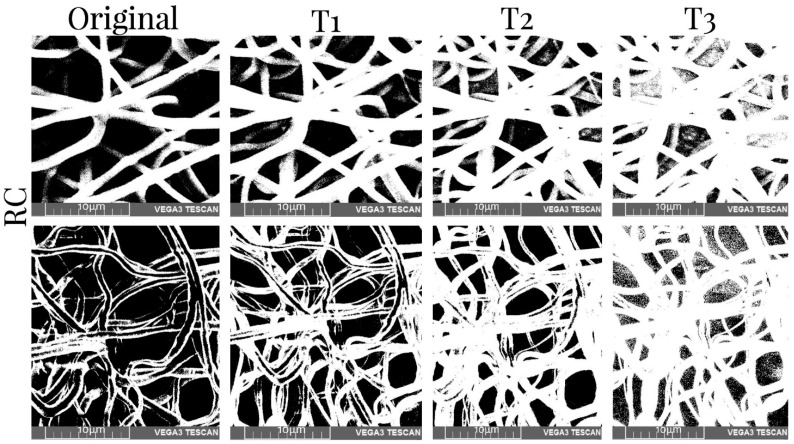
SEM images of the obtained RC45 scaffolds and binary images with three thresholds before (top row) and after mechanical tests (bottom row). T1–T3: threshold 1–3.

**Figure 7:**
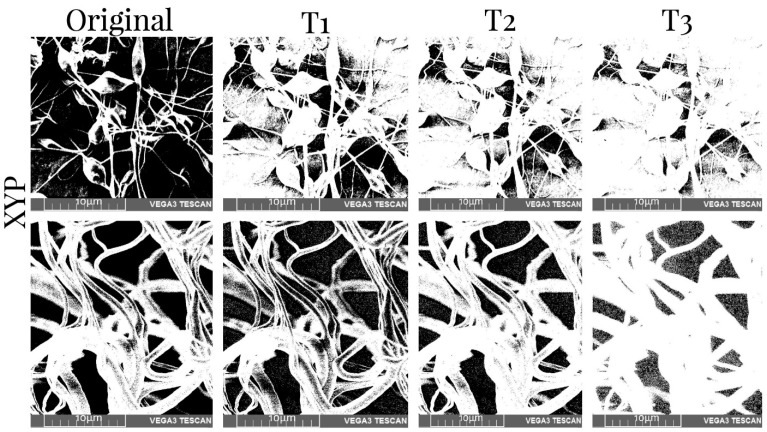
SEM images of the obtained XYP45 scaffolds and binary images with three thresholds before (top row) and after mechanical tests (bottom row). T1–T3: threshold 1–3.

**Figure 8:**
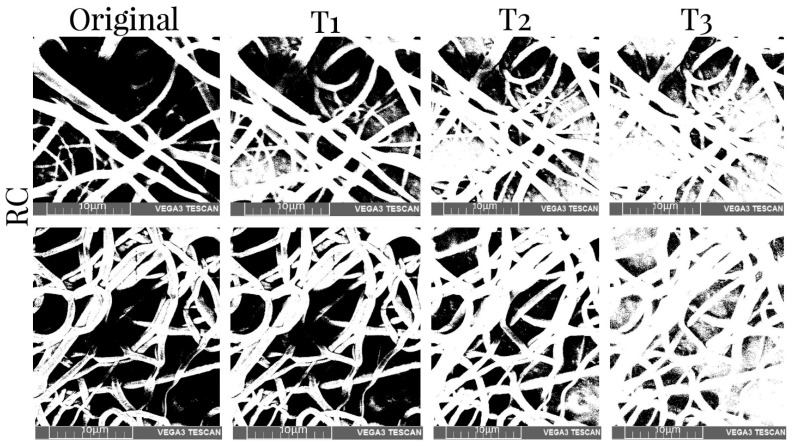
SEM images of the obtained RC90 scaffolds and binary images with three thresholds before mechanical tests (top row) and after mechanical tests (bottom row). T1–T3: threshold 1–3.

**Figure 9:**
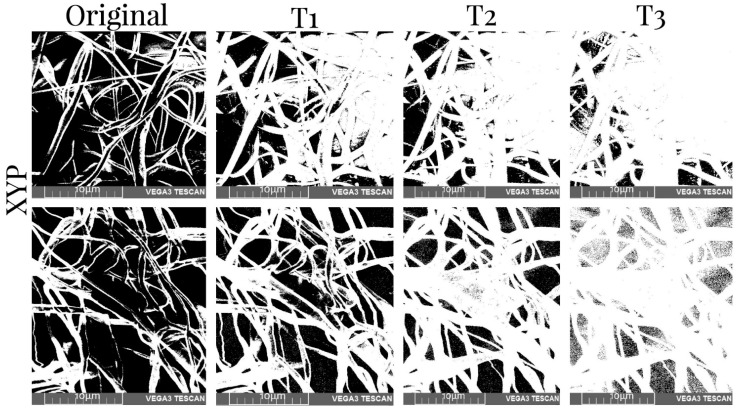
SEM images of the obtained XYP90 scaffolds and binary images with three thresholds before mechanical tests (top row) and after mechanical tests (bottom row). T1–T3: threshold 1–3.

Table 5 shows the values of the porosity measurements of binary images of different samples with various thresholds before and after mechanical tests. It was observed that the porosity was greater in the superficial layers of the scaffold and decreased with depth. On average, there was 11% less porosity per threshold. Additionally, the number of pores was reduced by approximately 10% when the XYP collector was used. The collection time showed no relationship with the porosity of the scaffolds. The binary SEM images of the three thresholds for the four samples produced using the RC and XYP as collectors with deposition times of 45 min and 90 min are shown in Figure 6, Figure 7, Figure 8 and Figure 9.

## 4. Discussion

The design and manufacture of nano/microfibrous membranes that function as scaffolds, mimic the ECM, and thus optimize tissue regeneration is one of the main challenges in TE and represents an alternative to the limited number of donors and the implications involved with the use of autologous grafts. Allografts may be capable of transmitting disease and eliciting host immune responses. A scaffold must be manufactured considering the porosity, the balance between hydrophilicity/hydrophobicity, the mechanical properties, the three-dimensional architecture, the biocompatibility, and the non-toxicity of its components. For its manufacturing, electrohydrodynamic techniques, such as electrospinning, are the most frequently used given their possibilities, versatility, low cost, and the simplicity and reproducibility of the process. The modification of parameters in the polymeric solution used and the process (voltage, solution flow, distance from the collector needle) and environmental conditions (temperature and RH), allows one to obtain scaffolds with particular characteristics in terms of their morphology, the dimensions and orientation of the fibers, their porosity, hydrophilicity, and their tensile strength for a specific use, especially in bone, musculoskeletal, cutaneous, cardiovascular, or neurological applications, among others [19,20,21,32]. Numerous studies have demonstrated the effect that the type of collector used has on the characteristics of the fibers and the scaffold itself. Multiple variants of RC and static flat collectors have traditionally been used [22,23,24,25]. More recently, the use of platforms with displacements in the X and Y axes has been introduced to facilitate the manufacturing of three-dimensional scaffolds with greater control over their shape and dimensions [33]. In this work, nano/microfibers were manufactured with PCL, a biodegradable polymer accepted by the FDA for surgical implants, drug delivery systems, and applications in TE and regenerative medicine [34]. To evaluate whether there were significant differences in the scaffolds produced on a RC versus XYP, all the parameters were standardized: the composition of the solution, the process variables, and the environmental conditions, including the displacement speed of the XYP in each axis with the angular velocity of the RC, although this implied an rpm much lower than the one normally used.

Determining whether there are differences in roughness is essential because it is considered a critical factor in cell adhesion, proliferation, and differentiation. To avoid compression deformations due to the deposition of several layers, the fibers used in the roughness tests were collected 30 s after their deposition. Although no significant differences were observed in the fibers obtained in the two types of collectors, the surface roughness of the XYP scaffolds showed a higher standard deviation than that of RC, especially the Ra value. This allows us to assume that the roughness of the scaffold deposited on the CR is more homogeneous due to the intrinsic rotation movement and the traction it exerts when picking up the fiber. High roughness favors osteogenic differentiation, neurite outgrowth, and Schwann cell proliferation, but impairs chondrogenic differentiation and endothelial function, to cite a few examples [33], hence the collecting type is an important consideration when scaffolds are to be used in a specific application.

Mechanical test results are affected by material composition, microscopic imperfections, the manufacturing process, the loading rate, and the temperature during testing [35]. The results showed that it is also necessary to consider the total deposition time when fabricating a scaffold by means of electrospinning. The results obtained using the two types of collectors showed that the collection time is an important parameter since, by increasing the deposition time, a greater number of fibers was generated on the surface, causing changes in the mechanical properties. That is, a greater presence of fibers produces a structure with greater mechanical resistance. When comparing the scaffolds, we determined that the increase in the elongation at break value when using RC was possibly due to the absence of pearls in the fibers obtained compared to those observed in XYP. The SEM images indicated that the RC fibers had a more homogeneous morphology in terms of the length of the fibers. The diameter of the fibers did not present great variation with respect to those observed in the XYP. Additionally, the area under the curve of the graphs (Figure 3) indicated that the fibers obtained via RC presented greater deformation due to the localized and directed shape as they fell into the collector. Scaffolds made in the RC system with a deposition time of 45 min had a significantly higher tensile strength than scaffolds made in XYP. This difference was not observed when the deposition time was 90 min. The higher tensile strength of the scaffolds obtained in RC correlated with their higher porosity. The larger spaces between the fibers allow the scaffolds to buffer stress effectively and slow down structural deterioration. This is essential in scaffolds designed for interfaces between soft and hard tissues (for example, in surgery to reconstruct ligaments or articular cartilage with their reattachment to bone tissue), in which the scaffold acts as a material developed to perform a gradual mechanical transfer between ECM of different properties [36]. RC usually produces aligned fibers, in the case of this work, due to the low angular velocity used, and scaffolds with randomly oriented fibers were obtained with both types of collectors, but with a higher number of pearls in the XYP. The presence of beads modifies the mechanical properties of the scaffolds and must be considered when using the mentioned interfaces [37].

In this study, our goal was to compare the type of collector, keeping the concentration of the polymer the same; therefore, it was not possible to analyze the influence of the amount of the polymer on the mechanical properties. On the other hand, in previous studies, they determined that, after increasing the concentration of PCL or the load of another component in the fibers, such as drugs and proteins, the tensile strength of the PCL nanofibrous membranes decreased notably [38,39]. It should be noted that the fibers produced by means of electrospinning exhibit a relatively smooth deposition process; therefore, collisions can occur with neighboring fibers that move in different directions. This can cause periodic blockages of larger-diameter fibers in which a microstructural failure appears to occur sequentially, involving a balance between localized strain in the direction of traction and anisotropic point junction that locally resists deformation [40]. This could explain why the scaffolds made in the RC90 system showed slightly higher Young’s modulus values compared to the scaffolds in the XYP90 system.

Electrospinning allows one to obtain fibers with diameters between micrometers and nanometers. The critical factors that determine the diameter of the fibers are the flow, the concentration of the polymer solution, and the voltage used [41]. When the concentration of PCL solutions is increased, the diameter of the fibers generally increases, although with this technique it is necessary to consider the other variables involved [42]. When comparing fibers deposited on a fixed-plate collector versus RC, the latter have a smaller diameter that decreases as the rotation speed (rpm) increases [43]. Differences in mechanical properties were observed with speeds greater than 640 rpm. In this work, due to the low angular velocity used, there were no significant differences between the diameters of the fibers deposited on RC versus XPY, independently of the deposition time. The low speed can also be considered an important factor regarding why differences in fiber diameters were not observed before and after the mechanical tests [44].

The wettability of surfaces depends on the chemical composition and the microgeometry of their roughness. Models made to predict the contact angle of fibers manufactured via electrospinning show that the concentration of the solution is the determining parameter [45]. For other authors, surface roughness is the critical factor and, in some polymeric fibers, changes in wettability can be achieved by modifying roughness, without the need for chemical changes [46]. When other parameters were studied, it was observed that PCL fibers can behave in a hydrophilic or hydrophobic character, depending on the solvent used [47]. However, these studies did not consider the type of collector used. When the fibers deposited on copper collectors (sheet versus mesh) were compared, it was observed that the fibers collected in the copper sheet had a greater diameter, less roughness, and showed a more hydrophilic behavior than those collected in the copper sheet [48]. In this work, significant differences were observed in the fibers collected in XYP versus RC when the deposition time was 45 min. We observed that the fibers manufactured on RC had lower contact angle values and therefore a more hydrophilic behavior, which is a critical factor in the biocompatibility and bioactivity of a material, since wettability generally favors adhesion and cell proliferation and increases biomineralization in engineering for bone tissues [49]. Although the scaffolds manufactured in XYP cannot be considered hydrophobic, their values were close to 90° and they could be used in applications in which cell adhesion must be controlled or inhibited, for example, to reduce bacterial contamination or the formation of biofilms on medical devices, since decreasing their wettability results in a deterioration of the stability of the bacterial colonies and favors their detachment [50].

The effect of the ECM in cell proliferation, gene expression, differentiation, and migration highlight its importance as a design parameter for scaffolds with specific applications. Scaffold designs with controlled mechanical properties, roughness, porosity, and hydrophilicity, among others, can have a great impact on improving the success of TE applications in various medical treatments.

## 5. Conclusions

The scaffolds fabricated and observed in this study showed significant differences in most of their characteristics—morphology, roughness, hydrophilicity, and mechanical properties. Regarding the diameter and porosity of the fibers, the results were similar between the scaffolds. It has been validated that when scaffolds are manufactured using the electrospinning technique, differences in important properties can be obtained, even when using the same solution and the same manufacturing parameters, merely by changing the type of collector in the process. Given that scaffolds must be designed to guarantee the adequate survival, proliferation, and migration of a certain cell type, in this study we analyzed how the variations in the characteristics of the fibers obtained were essential to defining their potential application. For example, scaffolds with high roughness can be used in bone regeneration, whereas those with minimal roughness could be used to mimic the tunica intima of blood vessels. Therefore, the implementation of new technologies such as 2D moving platforms in this technique presents multiple applications in the design of scaffolds applied to TE. An important advantage of the XYP approach is the control of the shape of the scaffold produced. This is the first study to date, to our knowledge, that has compared the effects of electrospinning on the characteristics of the fibers and scaffolds while utilizing both a rotating collector and a displacement platform in the X-Y axes.

## Figures and Tables

**Table 1:** Surface roughness of the samples collected using the RC and XYP.

Collector Type		Root Mean Square (Rms) (nm)	Arithmetical Average (Ra) (nm)
**RC**	**Mean** **(nm)**	51.47	42.72
	**SD**	15.33	12.13
**XYP**	**Mean** **(nm)**	36.94	38.25
	**SD**	15.62	23.72

**Table 2:** Mechanical testing results of PCL membranes.

Collector Type	Deposition Time	Sample	Max_Force N	Max_Disp mm	Max_Stress N/mm^2^ MPa	Max_Strain %
RC	45 min	Mean value	0.18	105.06	0.39	114.91
		SD	0.02	19.39	0.05	25.44
	90 min	Mean value	0.19	118.87	0.41	119.88
		SD	0.04	13.83	0.08	21.05
XYP	45 min	Mean value	0.06	80.53	0.12	70.98
		SD	0.01	32.99	0.02	49.14
	90 min	Mean value	0.15	77.47	0.33	75.87
		SD	0.02	24.51	0.03	24.95

Abbreviations: Max force, maximum force; Max Disp, maximum disposition; Max Stress, maximum Stress; Max Strain, maximum strain; min, minutes.

**Table 3:** Diameter and statistics of different fibers of the samples collected using RC and XYP before and after mechanical testing.

Collector Type	Deposition Time	Diameter (nm)			
		Mean (nm)		SD	
		Before	After	Before	After
**RC**	RC45	1819	1147	549	424
	RC90	1386	1267	674	404
**XYP**	XYP45	548	1357	502	672
	XYP90	1472	1005	846	820

**Table 4:** Contact angles according to collector type.

Deposition Time		Contact Angle
RC45	**Mean**	51.95°
	**SD**	1.80
RC90	**Mean**	58.19°
	**SD**	6.66
XYP45	**Mean**	80.52°
	**SD**	3.87
XYP90	**Mean**	74.77°
	**SD**	15.63

**Table 5:** Porosity measurements of binary images of different samples with various thresholds before and after mechanical tests.

Collector Type	Deposition Time	Sample Type	T1%	T2%	T3%
**RC**	45 min	Before	38.19	25.95	12.71
		After	48.09	34.97	22.36
	90 min	Before	39.32	26.39	17.11
		After	29.29	19.88	15.68
**XYP**	45 min	Before	28.06	18.89	9.26
		After	47.80	31.42	15.45
	90 min	Before	29.42	19.47	9.66
		After	46.30	28.34	10.16

## Data Availability

Not applicable.
